# Evaluation of the BD urine culture application for artificial intelligence-assisted urine culture interpretation

**DOI:** 10.1128/jcm.00270-26

**Published:** 2026-06-22

**Authors:** Petrus van der Walt, Prenilla Naidu, Silvana Avoledo, Cecilia Mak, Francene Starko

**Affiliations:** 1 Alberta Precision Laboratories 113196 , Edmonton, Alberta, Canada; 2 Department of Laboratory Medicine and Pathology, University of Alberta 3158, Edmonton, Alberta, Canada; Children's Hospital Los Angeles, Los Angeles, California, USA

**Keywords:** artificial intelligence, automation, urine culture

## Abstract

Artificial intelligence-based culture reading tools can potentially accelerate reading, improving reporting consistency in image-based interpretation, which is relevant for high-volume urine cultures. This study evaluated the BD Kiestra Urine Culture Application (UCA) in a centralized microbiology laboratory. Consecutive midstream and indwelling catheter urine specimens inoculated onto BD BBL CHROMagar Orientation were analyzed by the UCA. Growth score output at different reporting thresholds was evaluated, along with expert rule-based categories constructed using the app’s growth score, purity score, and presumptive identification parameters, with blinded technologist image reads as the comparator. Of 1,304 cultures, 1,251 were evaluable for growth score comparison, and 1,239 for expert-rule assessment. The exact agreement across four growth categories (<10^5^, 10^5^, 10^6^, ≥10^7^ CFU/L) was 86.2%. Using a <10^6^ CFU/L triage threshold, the overall agreement was 95.9%, with positive and negative percent agreement of 89.9% and 98.9%, respectively. In this dataset, 30.5% of cultures met this threshold for potential auto-release or batch-review. For presumptive identification in ≥10^7^ CFU/L pure or predominant cultures, UCA performed best for *E. coli* (PPA 98.9%). For *Enterococcus* spp., PPA was 89.5%, but PPV was low (25.4%), reflecting frequent false-positive presumptive calls. UCA performed well at clinically relevant thresholds and could meaningfully reduce the burden of manual reading. Most discrepancies reflected differences between total colony count outputs and morphotype-based laboratory quantitation conventions, along with missed subtle growth. Expert-rule category discrepancies were primarily related to differences in purity output and presumptive identification, particularly fine light-blue growth being called presumptive *Enterococcus* spp., highlighting a priority area for improvement.

**IMPORTANCE:**

Urine cultures are among the most frequently ordered culture tests in clinical microbiology. Timely, consistent plate interpretation impacts both patient care and laboratory capacity. Digital imaging of culture plates facilitates the application of artificial intelligence to urine culture specimens, but laboratories need clear evidence of where these tools perform reliably and where their outputs require caution. This study shows how an automated urine culture reading application can support safe triage of cultures with little or no growth, helping technologists focus on specimens that likely require workup and clinical action. At the same time, it highlights an important limitation: organism labeling can be vulnerable to look-alike growth patterns. In our dataset, fine or hazy, light-blue growth that was not typical of *Enterococcus* spp. was classified by the application as presumptive *Enterococcus* spp., a mismatch that could affect downstream decisions if not recognized. These findings reinforce that local validation and human oversight remain essential, even for common uropathogens, such as *Escherichia coli*.

## INTRODUCTION

Urinary tract infections (UTIs) are among the most common bacterial infections encountered in both community and hospital settings, and they contribute substantially to health system burden and antibiotic exposure (1, 2). Urine culture is an essential laboratory test, but it is not, on its own, diagnostic of urinary tract infection. Culture findings are semi-quantitative and inherently interpretive because reported significance depends on the quality of specimen collection, bacterial load, technologist-to-technologist variation, and the clinical context. Asymptomatic bacteriuria is a common finding, and outside of specific indications (for example pregnancy and selected urologic procedures), treatment is generally not recommended (3). Inappropriate urine culture ordering and subsequent treatment of asymptomatic bacteriuria are well-recognized drivers of unnecessary antimicrobial prescribing and avoidable harms, making diagnostic stewardship for urine cultures an important quality and patient safety priority (3, 4).

From a microbiology laboratory operational perspective, urine cultures are among the highest-volume workflows and require substantial laboratory resources for processing, plate reading, and follow-up work (5). This has driven adoption of total laboratory automation that standardizes inoculation, incubation, and enables image-based reading (5). Digital imaging also provides the foundation for algorithm-assisted interpretation, including automated triage of no growth or minimal growth cultures and prioritization of plates requiring technologist review (6, 7).

Chromogenic urine media, such as BD BBL CHROMagar Orientation, were developed to support primary isolation while improving visual differentiation of common uropathogens, simplifying plate interpretation, enabling presumptive identification, and recognition of mixed growth, and streamlining downstream workup. The medium incorporates chromogenic substrates that detect organism-associated enzyme activity and produce characteristic colony and halo colors, and it supports presumptive calls for a defined set of common uropathogen targets, including *Escherichia coli*, *Enterococcus* spp., the *Klebsiella-Enterobacter-Serratia* (KES) group, the *Proteus-Morganella-Providencia* (PMP) group, *Streptococcus agalactiae*, and *Staphylococcus saprophyticus* (8, 9).

The BD Kiestra Urine Culture Application (UCA) is an artificial intelligence solution for the interpretation of urine culture plate images and provides outputs, such as growth score, purity, and presumptive identification. Early evaluations have reported high concordance with expert image-based reads and suggest potential efficiency gains (10). However, performance and clinical utility depend on local practice, particularly because laboratory conventions for semi-quantitation and mixed growth interpretation may differ from application-defined scoring approaches.

The aim of this study was to evaluate the UCA on chromogenic urine culture plate images within a routine digital microbiology workflow. We assessed performance of different reporting thresholds to facilitate triage of cultures, and evaluated whether UCA outputs, including growth score, purity, and presumptive identification, can be combined into expert-rule categories to generate practical worklists within the BD Synapsys workflow.

## MATERIALS AND METHODS

We performed a prospective, single-center method evaluation of the UCA in a centralized clinical microbiology laboratory that processes more than 25,000 urine cultures per month.

### Specimens and inclusion criteria

Consecutive urine specimens were enrolled over a defined 24-h period to capture a representative range of routine submissions. Inclusion criteria were preserved urine submitted in BD Vacutainer Culture and Sensitivity (C & S) Preservative Tubes and processed within 48 h of collection. Only midstream and indwelling catheter urine specimens were included. Other urine sources were excluded from this evaluation.

### Standard laboratory workflow

Specimens were inoculated onto BD BBL CHROMagar Orientation plates using the BD Kiestra InoqulA, with an automated 10-µL inoculum and a standardized streak pattern (Pattern 4, zig-zag with progressive narrowing using a magnetic bead). Plates were incubated aerobically at 35°C ± 1°C within the BD Kiestra ReadA smart incubators. Routine interpretation was performed from plate images at 21 h. Each visible colony morphotype was assessed separately, and semi-quantitation was assigned per morphotype using our reporting categories: 10^5^ CFU/L (10^2^ CFU/mL) for 1 to 9 colonies, 10^6^ CFU/L (10^3^ CFU/mL) for 10 to 99 colonies, and ≥10^7^ CFU/L (≥10^4^ CFU/mL) for ≥100 colonies. Our laboratory caps reporting at ≥10^7^ CFU/L, since counting to 1,000 colonies or calling confluent growth is time-consuming and subjective, and it does not change subsequent interpretation and workup for common uropathogens. For cultures with <10^6^ CFU/L, a final report is issued. For cultures with 10^6^ CFU/L or higher, the extent of additional workup and reporting follows established laboratory SOPs aligned with established guidance (11).

### Urine culture application

The UCA is an *in vitro* diagnostic software program that analyzes a time-series of images of urine culture samples processed, inoculated, and incubated on the BD Kiestra total laboratory automation system, and it runs within the BD Synapsys Informatics Solution Application (version 4.20.2021.27208). Its use is limited to BD-claimed media and in our implementation, it was applied to BD BBL CHROMagar Orientation plates. Plates were inoculated with 10-µL urine, incubated, and imaged at 2, 11, and 21 h. If all quality assurance prerequisites are met, the culture is set to Ready for Reading.

For each plate, the UCA generates three output parameters: growth score, purity score, and presumptive identification.

Growth score is colony-count based. With a 10-µL inoculum, each colony corresponds to 10^5^ CFU/L (100 CFU/mL), and growth is binned as follows: 0 colonies, <10^5^ CFU/L (<10^2^ CFU/mL); 1 to 9 colonies, 10^5^ to <10^6^ CFU/L (10^2^ to <10^3^ CFU/mL); 10 to 99 colonies, 10^6^ to <10^7^ CFU/L (10^3^ to <10^4^ CFU/mL); 100 to 999 colonies, 10^7^ to <10^8^ CFU/L (10^4^ to <10^5^ CFU/mL); and ≥1,000 colonies or confluent growth, ≥10^8^ CFU/L (≥10^5^ CFU/mL).

Purity is output as Pure when ≥99% of observable growth is classified as one colony morphotype, Predominant when two or more colony morphotypes are present but one comprises 90% to 99% of observable growth, and Complex when mixed growth does not meet criteria for Pure or Predominant.

Presumptive identification is generated from detected colonies on BD BBL CHROMagar Orientation when the purity result is Pure or Predominant. Presumptive identification categories include *E. coli*, *Enterococcus* spp., KES group, PMP group, *S. agalactiae*, *S. saprophyticus*, and Other. No presumptive identification is assigned when the purity result is Complex.

UCA output validity depends on meeting predefined pre-analytic and imaging requirements; for example, completion of the three-image time series within allowable time windows. If requirements are not met, no reportable parameter output is generated and the culture is routed for manual review (12).

### Alignment of UCA outputs with laboratory reporting conventions

We aligned UCA outputs with our laboratory’s routine urine culture reporting conventions. The UCA outputs a growth score based on total observable colony count, regardless of the presence of different colony morphologies. In contrast, our routine reporting assigns the semi-quantitative growth category to the single morphotype with the highest growth, since interpretation is organism-specific rather than based on total or summed growth. Our reporting convention also limits semi-quantitation at ≥^7^ CFU/L, as mentioned above. The UCA purity score was interpreted using the manufacturer definitions described above. Our reporting assigns “predominant” only when the highest-growth morphotype is at least one semi-quantitative category higher than the next morphotype. UCA outputs were interpreted using these local conventions, recognizing that this will result in predictable differences compared with morphotype-based reporting.

### Comparator method

We compared UCA-assisted interpretation with routine image-based culture interpretation using our usual reporting conventions. Plate images were imported into the BD Colony Information System (CIS), a BD-provided image review tool that allows technologists to review images outside the BD Synapsys user interface. These were the same plate images used for routine clinical reporting; however, comparator reads were performed by different technologists who were not involved in the original clinical reporting workflow and had not previously reviewed the study images. Reading in the CIS tool ensured comparator reads were not influenced by UCA image labels displayed during routine reading in BD Synapsys.

All technologists performing comparator reads were trained on the CIS prior to study initiation. Two independent technologists assessed plate images using our standard reporting conventions and were blinded to each other’s interpretations and to the UCA outputs during the primary read. When the two readers did not agree, the case was adjudicated by a third reader to establish the final comparator result used for analysis, with adjudication performed in the CIS tool.

### Statistical analysis

Performance was summarized using confusion matrices for each evaluated output. Overall categorical agreement (OA), positive percent agreement (PPA), and negative percent agreement (NPA) were calculated as the primary agreement metrics using standard methods. Positive predictive value (PPV) and negative predictive value (NPV) were also calculated but were used only to contextualize the practical impact of results within the evaluated workflow, recognizing that the comparator was blinded technologist image interpretation rather than an independent clinical gold standard (13). Then, 95% confidence intervals were calculated using the Wilson method (14).

### Discrepancy analysis

Discrepancy analyses were performed by an experienced supervisory technologist and a medical microbiologist. Culture images were re-reviewed, and a detailed plate description was recorded, including colony counts for each morphotype present. Discrepancies were not assessed in real time; therefore, the original samples and plates were not available for manual rework or direct plate review. These descriptions were used to characterize discrepancy patterns and to apply UCA definitions for growth score, purity, and presumptive identification post hoc. This enabled an updated growth-score confusion matrix, along with updated category and threshold analyses, to describe definitional differences between the UCA and our laboratory reporting conventions.

## RESULTS

A total of 1,304 cultures were imported into the CIS for comparator reading. A total of 53 were excluded from growth score analysis because no UCA output was generated. These were due to predefined requirements not being met, majority being incubation interruption events or incomplete imaging time points. This resulted in 1,251 cultures available for growth score evaluation. An additional 12 cultures were excluded due to incomplete comparator data for purity and preliminary identification parameters utilized in the expert-rule category analysis, leaving 1,239 cultures for expert-rule evaluation.

Growth score agreement across the four reporting categories (<10^5^, 10^5^, 10^6^, ≥10^7^ CFU/L) is shown in the confusion matrix in Table 1. Exact agreement occurred in 1,078 of 1,251 cultures (86.2%). Discrepancies were largely between adjacent categories. The most common discrepancies were UCA ≥10^7^ CFU/L vs comparator 10^6^ CFU/L (*n* = 75) and UCA 10^6^ CFU/L vs comparator 10^5^ CFU/L (*n* = 40), followed by UCA 10^5 CFU/L vs comparator <10^5 CFU/L (*n* = 19) and comparator 10^5^ CFU/L vs UCA <10^5^ CFU/L (*n* = 18) (Table 1). Threshold-based performance is summarized in Table 2. For the <10^5^ CFU/L threshold, overall agreement was 96.8% (1,211/1,251), with PPA 89.9% (85.0–93.3) and NPA 98.2% (97.2–98.8). For the <10^6^ CFU/L threshold, the overall agreement was 95.9% (1,200/1,251), with PPA 89.9% (86.6–92.4) and NPA 98.9% (98.0–99.4). For the <10^7^ CFU/L threshold, the overall agreement was 93.0% (1,164/1,251), with PPA 89.1% (86.5–91.2) and NPA 97.9% (96.3–98.8) (Table 2). Applying the <10^6^ CFU/L threshold would have assigned 382 of 1,251 cultures (30.5%) to the no-growth or low-growth category eligible for potential auto-release or batch review.

**TABLE 1:** Growth score confusion matrix (CFU/L)

		Technologist			
		<10^5^	10^5^	10^6^	≥10^7^
UCA	<10^5^	186	18	1	0
	10^5^	19	150	6	2
	10^6^	2	40	188	10
	≥10^7^	0	0	75	554

**TABLE 2:** Growth score agreement by threshold^*a*^

Growth score Threshold	TP	FP	FN	TN	OA (n[%])	PPA (%[95% CI])	NPA (%[95% CI])
<10^5^ cfu/L	186	19	21	1025	1211/1251 (96.8)	89.9 (85-93.3)	98.2 (97.2-98.8)
<10^6^ cfu/L	373	9	42	827	1200/1251 (95.9)	89.9 (86.6-92.4)	98.9 (98-99.4)
<10^7^ cfu/L	610	12	75	554	1164/1251 (93)	89.1 (86.5-91.2)	97.9 (96.3-98.8)

^*a*^ TP, true positive; FP, false positive; FN, false negative; TN, true negative; OA, overall agreement; PPA, positive percent agreement; NPA, negative percent agreement; CI, confidence interval.

After discrepancy analysis and post hoc application of UCA growth score definitions to the image-based plate descriptions, agreement improved (Table S1 and S2). Exact agreement increased to 1,180 of 1,251 cultures (94.3%) (Table S1). Threshold-based performance also improved (Table S2). For the <10^5^ CFU/L threshold, the overall agreement was 96.9% (1,212/1,251), with PPA 90.3% (85.5–93.6) and NPA 98.2% (97.2–98.8). For the <10^6^ CFU/L threshold, the overall agreement increased to 98.6% (1,234/1,251), with PPA 97.9% (95.9–98.9) and NPA 99.0% (98.0–99.5). For the <10^7^ CFU/L threshold, the overall agreement was 98.2% (1,229/1,251), with PPA 98.7% (97.5–99.3) and NPA 97.8% (96.3–98.7) (Table S2).

Performance of prespecified expert rule categories is summarized in Table 3 (*n* = 1,239). For ≥10^7^ CFU/L, Pure or Predominant, *E. coli*, PPA was 98.9% with PPV 69.5%. For ≥10^7^ CFU/L, Pure or Predominant, *Enterococcus* spp., PPA was 89.5% with PPV 25.4%, with a higher false-positive rate despite high NPV (99.6%). For ≥10^7^ CFU/L, Pure or Predominant, KES (*Klebsiella* spp., *Enterobacter* spp., *Serratia* spp.), PPA was 100% with PPV 41.9%. Categories incorporating Complex purity showed lower agreement, including 10^6^ CFU/L, Complex (PPA 56.4%, PPV 50.8%) and ≥10^7^ CFU/L, Complex (PPA 54.5%, PPV 41.4%).

**TABLE 3:** Expert rule category agreement and performance^*a*^

Expert rule category (one-vs-rest)	TP	FP	FN	TN	OA (*n* [%])	PPA (% [95% CI])	NPA (% [95% CI])
10^6^, Complex	97	94	75	973	1,070/1,239 (86.4)	56.4 (48.9–63.6)	91.2 (89.3–92.7)
≥10^7^, Pure, Predominant, *E. coli*	89	39	1	1,110	1,199/1,239 (96.8)	98.9 (94–99.8)	96.6 (95.4–97.5)
≥10^7^, Pure, Predominant, *Enterococcus* spp.	34	100	4	1,101	1,135/1,239 (91.6)	89.5 (75.9–95.8)	91.7 (90–93.1)
≥10^7^, Pure, Predominant, KES (*Klebsiella*, *Enterobacter*, *Serratia*)	18	25	0	1,196	1,214/1,239 (98)	100 (82.4–100)	98 (97–98.6)
≥10^7^, Complex	121	171	101	846	967/1,239 (78)	54.5 (47.9–60.9)	83.2 (80.8–85.4)

^*a*^ TP, true positive. FP, false positive. FN, false negative. TN, true negative. OA, overall agreement. PPA, positive percent agreement. NPA, negative percent agreement. CI, confidence interval.

**Fig 1:**
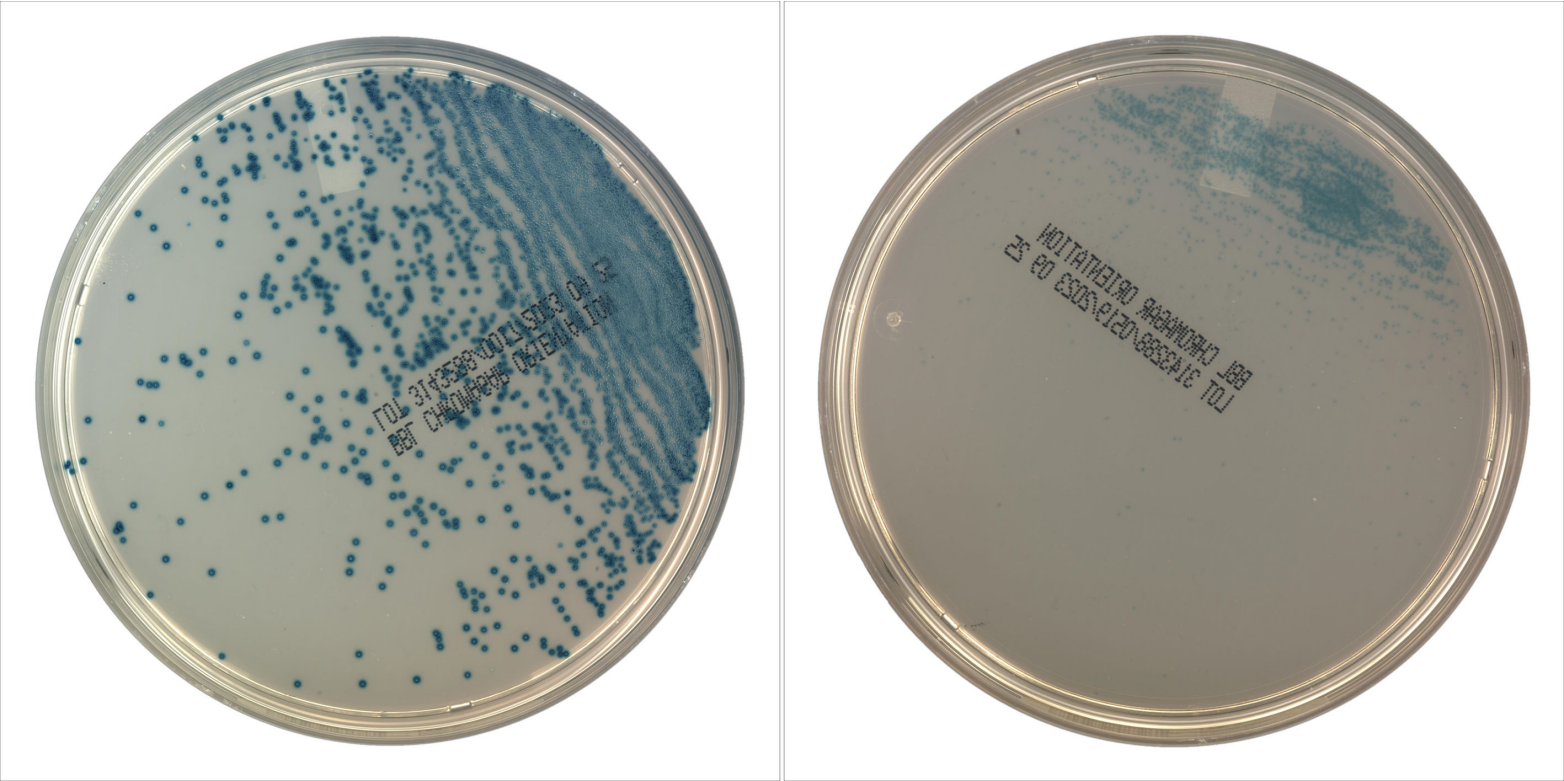
Representative colony morphology on BD BBL CHROMagar Orientation. The left image shows *Enterococcus* spp., characterized by small blue to blue-green colonies, often with a halo. The right image shows non-*Enterococcus* growth, characterized by fine, hazy, light-blue growth.

During discrepancy analysis, many discrepancies were resolved and were due to comparator reading issues, for example technologists not recognizing tiny, fine, or faint colonies on the initial image read, rather than UCA behavior. Table 4 summarizes the main UCA-related and definition-related discrepancy patterns observed during image discrepancy analysis. For threshold analyses (<10^5^, <10^6^, and <10^7^ CFU/L), discrepancies were mostly related to definitional differences between plate level and morphotype-based interpretation (see Materials and Methods). This was most apparent in mixed growth, where the UCA assigns a plate-level growth category based on total colonies across morphotypes, while routine reporting assigns growth based on the highest-growth single morphotype. Additional threshold discrepancies were due to missed subtle growth and, less commonly, UCA recognition of non-colony features such as bubbles or dust (Table 4). For expert rule categories, discrepancies were mostly related to differences in purity assignments and presumptive identification, particularly fine, hazy, light-blue growth not typical of *Enterococcus* spp. being interpreted as presumptive *Enterococcus* spp. (Table 4). Representative images of a pure *Enterococcus* spp. culture and fine, light-blue growth not typical of *Enterococcus* spp. are shown in Fig. 1.

**TABLE 4:** Summary of observations during discrepancy analysis

Category	Discrepancy description	*N*
Threshold analysis (<10^5^ CFU/L, <10^6^ CFU/L, and <10^7^ CFU/L)	Difference between UCA total plate colony count and local morphotype-based reporting convention Fine growth not recognized or under-counted by UCA Bubbles, dust, or other non-colony features likely interpreted as growth by UCA One or two tiny colonies not recognized by UCA UCA discrepancy reason unclear	58
		28
		30
		18
		4
Growth score	Difference between UCA total plate colony count and local morphotype-based reporting convention Fine growth not recognized or under-counted by UCA Bubbles or other non-colony features likely interpreted as growth by UCA UCA discrepancy reason unclear	93
		13
		2
		14
Purity	Difference between UCA purity definitions and local mixed-growth interpretation Two or more distinct non-blue colony morphologies present on the same plate, but interpreted by UCA as pure Two or more distinct blue colony morphologies present on the same plate, but interpreted by UCA as pure Fine growth not recognized by UCA as pure UCA discrepancy reason unclear	38
		48
		64
		61
		149
Presumptive identification	Fine light blue growth interpreted by UCA as presumptive *Enterococcus* spp. UCA discrepancy reason unclear	62
		4

## DISCUSSION

We evaluated the BD Kiestra Urine Culture Application on chromogenic urine culture plate images to support triage of cultures with no growth or very little growth (<10^6^ CFU/L) for reporting. We also assessed whether the UCA outputs, growth score, purity, and presumptive identification can be combined into expert-rule categories that generate practical worklists within the BD Synapsys workflow.

Overall, we found that UCA is reliable for threshold-based triage in our digital reading workflow, both at the <10^6^ CFU/L and <10^7^ CFU/L decision thresholds. The <10^6^ CFU/L corresponds to no growth or fewer than 10 visible colonies. This amount of growth is not typically detectable if urine cultures are processed using a 1-µL loop compared with the BD Kiestra InoqulA 10-µL inoculation volume. This is also the threshold by which automated release of results can occur unless a more conservative batch review workflow is preferred.

False-positive results were infrequent at the <10^6^ CFU/L threshold, and 8 out of 9 were due to ≥10^7^ CFU/L growth of very fine growth. However, this app behavior is likely to be of limited clinical significance, as this type of growth morphology is indicative of urogenital flora contamination. Of the 42 false-negative results, 20 were attributable to a difference in definition between our laboratory convention of morphotype-based quantitation and the UCA total colony count growth scoring approach, and a further 14 were resolved on re-review as missed tiny, fine, or faint colonies on the initial technologist assessment.

Applying the <10^6^ CFU/L rule would have routed 382/1,251 (30.5%) cultures into an auto-release or batch-review worklist pathway for no growth or low growth, consistent with our local experience where about 25% to 30% of routine urine cultures are reported in these categories. In a routine implementation, the effect on workload would depend on local urine culture volume, reporting rules, and staffing model. False negatives at this threshold are mainly a workflow consideration rather than a patient safety issue because they simply default to the manual worklist for technologist review. In that context, the 95.2% NPV suggests that the incremental effect on workload is small, with only about 1 in 20 cultures on the manual list being reported as no growth or 10^5^.

At the <10^7^ CFU/L (<100 colonies) cutoff, threshold performance remained strong (OA 93%, PPA 89.1%, NPA 97.9%, PPV 98.1%, NPV 88.1%) and would have routed 622/1,251 (49.7%) cultures into the batch-review worklist pathway. As above, most discrepancies reflected the same total colony count versus morphotype-based quantitation difference. At discrepancy analysis, 59 discrepancies were attributable to growth not being recognized by the technologist reading the culture, including 22 instances of tiny, fine, or faint colonies and 37 instances of readily visible colonies not initially identified, supporting improved reproducibility of growth detection and quantitation.

Our threshold findings are consistent with prior automated plate-reading studies when you align them to comparable decision cutoffs. The UCA proof-of-concept by Croxatto et al. evaluated growth detection at 100 CFU/mL (that is, <10^5^ CFU/L or no growth) and reported strong performance (sensitivity 97.1%, specificity 93.6%) (15), which is similar to our <10^5^ CFU/L threshold results, but in routine reporting, the more clinically relevant triage boundaries are <10^6^ and <10^7^ CFU/L. For direct comparison, we used the orientation-medium confusion matrix in the supplementary appendix from Uwamino et al. and collapsed colony-count bins to these same cutoffs; the derived performance was OA 97.3%, PPA 96.4%, and NPA 98.3% at <10^6^ CFU/L and OA 98.5%, PPA 98.5%, and NPA 98.4% at <10^7^ CFU/L (10), compared with our OA 95.9%, PPA 89.9%, and NPA 98.9%, and OA 93.0%, PPA 89.1%, and NPA 97.9% at the same thresholds. Faron et al. reported WASPLab binary performance using a 10,000 CFU/mL cutoff (that is, <10^7^ CFU/L), with very high sensitivity (99.8%) and lower initial specificity that improved after discrepancy analysis, largely driven by microcolonies near the cutoff (6). Chiu et al. evaluated APAS using rule-based sorting into a small number of actionable bins, but the results are presented as growth-pattern agreement rather than threshold-based PPA and NPA at fixed CFU cutoffs, which limits direct comparison to our threshold analyses (7).

For organism-level expert rules at ≥10^7^ CFU/L, we focused on the three most common presumptive identification categories in our dataset: *E. coli*, *Enterococcus* spp., and KES. The *E. coli* expert rule set performed best and is useful for prioritization, but most discrepancies were due to cultures being incorrectly interpreted as pure, despite additional morphotypes on the plate. Cultures with more than one morphology still require subculture to obtain a pure isolate, so this category cannot be used to reflexively order susceptibility testing directly from the primary plate. An improvement for future versions would be more reliable purity score performance to adequately support reflex workup decisions. The KES rule performed generally well, but PPV remained modest, so it is best suited for prioritization rather than reflex workup.

In contrast, the *Enterococcus* spp. expert rule set performed poorly, and the pattern of discrepancies differed from the growth-score threshold discrepancies described above. Most *Enterococcus* spp. expert-rule discrepancies were driven by fine, light-blue growth not typical of *Enterococcus* spp. being interpreted by the UCA as presumptive *Enterococcus* spp. This growth was visually distinct from typical small blue to blue-green *Enterococcus*-like colonies on CHROMagar Orientation. Additional discrepancies were associated with multiple blue colony morphologies in significant amounts, consistent with mixed cultures being classified as pure or predominant. This misclassification pattern was not reported in prior UCA proof-of-concept and evaluation studies (10, 15), and we suspect that this fine, light-blue growth, suggestive of *Lactobacillus*-like urogenital flora, may have been underrepresented in the datasets used to train and evaluate the presumptive identification parameter.

Our study has several key strengths. We evaluated the UCA independently in a high-volume, total laboratory automation setting using a chromogenic medium that is routinely used for urine culture workup. Most of the key published performance evaluations used for comparison are industry-sponsored, and our study provides an external assessment in a routine clinical workflow. Because UCA output parameters are visible to the reading technologist and cannot be turned off in the Synapsys reading interface, we reduced incorporation bias by using dual blinded reads with arbitration in a dedicated evaluation tool. We report UCA performance in laboratory terms that map to routine reporting and workup decisions, including threshold-based triage and expert-rule categories derived from growth score, purity score, and presumptive identification. We also describe why discrepancies occur, including definitional differences between total colony count and morphotype-based growth scoring, and organism-level misclassification patterns, such as fine, light-blue growth not typical of *Enterococcus* spp. being called presumptive *Enterococcus* spp., which provides practical guidance for implementation and for future dataset development.

This study has several limitations. It was a single-center study, and organism distribution and practice patterns may differ from other laboratories, with some UCA presumptive identification categories underrepresented in our dataset. We assessed growth score performance on a single chromogenic medium, and performance may differ on other media used in urine culture workflows. Our comparator was digital image interpretation, and we did not review physical plates or perform additional testing from the original specimen, which reflects routine practice in a digital workflow but limits discrepancy analysis. Generalizability is most appropriate to laboratories using the same generation of inoculation and imaging systems with similar media and streaking patterns, given the narrow specification of the application. Finally, we did not measure formal operational metrics during the evaluation period due to operational constraints.

### Conclusion

This study demonstrates that the BD Kiestra UCA performs well for threshold-based triage of chromogenic urine cultures, particularly at the clinically relevant <10^6^ CFU/L and <10^7^ CFU/L decision thresholds. By supporting auto-release or batch review of <10^6^ CFU/L growth, the UCA could improve efficiency by reducing the time technologists spend reporting negative or low-growth urine cultures. Future prospective implementation studies are required to quantify actual efficiency gains. Applying a consistent AI algorithm to every image may also improve reproducibility of growth detection and semi-quantitation and reduce reader-to-reader variability inherent to manual interpretation (6, 10). In contrast, organism-level expert rules were less reliable, with performance limited by purity score assignments and presumptive identification, most notably fine, light-blue growth not typical of *Enterococcus* spp. being interpreted as presumptive *Enterococcus* spp., highlighting a priority area for future improvement before reflex organism identification-directed workup can be safely automated.

## SUPPLEMENTAL MATERIAL

Tables S1 and S2 jcm.00270-26-s0001.docx Post discrepancy analysis growth score data.

ASM does not own the copyrights to Supplemental Material that may be linked to, or accessed through, an article. The authors have granted ASM a non-exclusive, world-wide license to publish the Supplemental Material files. Please contact the corresponding author directly for reuse.
