# Supplementary material for: Evaluation of the BD urine culture application for artificial intelligence-assisted urine culture interpretation
Source: J Clin Microbiol. 2026 Jun 22;64(8):e00270-26. doi: 10.1128/jcm.00270-26 (PMC13463778; doi:10.1128/jcm.00270-26)
Supplement: Supplementary file 1 [file jcm.00270-26-s0001.docx]

**Supplementary Tables**

| Supplementary Table 1: Growth score confusion matrix post discrepancy analysis and UCA definition application (cfu/L) | | | | | |
| --- | --- | --- | --- | --- | --- |
|  |  | Technologist | | | |
|  |  | **<10^5** | **10^5** | **10^6** | **≥10^7** |
| UCA | **<10^5** | 186 | 18 | 1 | 0 |
|  | **10^5** | 18 | 151 | 4 | 4 |
|  | **10^6** | 2 | 6 | 222 | 10 |
|  | **≥10^7** | 0 | 0 | 8 | 621 |

| Supplementary Table 2: Growth score agreement by threshold post discrepancy analysis and UCA definition application | | | | | | | |
| --- | --- | --- | --- | --- | --- | --- | --- |
| **Growth score Threshold** | **TP** | **FP** | **FN** | **TN** | **OA  (*n*[%])** | **PPA  (%[95% CI])** | **NPA  (%[95% CI])** |
| **<10^5 cfu/L** | 186 | 19 | 20 | 1026 | 1212/1251 (96.9) | 90.3  (85.5–93.6) | 98.2 (97.2–98.8) |
| **<10^6 cfu/L** | 373 | 9 | 8 | 861 | 1234/1251 (98.6) | 97.9 (95.9–98.9) | 99  (98–99.5) |
| **<10^7 cfu/L** | 608 | 14 | 8 | 621 | 1229/1251 (98.2) | 98.7 (97.5–99.3) | 97.8 (96.3–98.7) |
| Abbreviations: TP, true positive. FP, false positive. FN, false negative. TN, true negative. OA, overall agreement. PPA, positive percent agreement. NPA, negative percent agreement. CI, confidence interval. | | | | | | | |
